# Resampling-based tests for Lasso in genome-wide association studies

**DOI:** 10.1186/s12863-017-0533-3

**Published:** 2017-07-24

**Authors:** Jaron Arbet, Matt McGue, Snigdhansu Chatterjee, Saonli Basu

**Affiliations:** 1 Division of Biostatistics, School of Public Health, University of Minnesota, Minneapolis, 55455 USA; 2 Department of Psychology, University of Minnesota, Minneapolis, 55455 USA; 3 Department of Statistics, University of Minnesota, Minneapolis, 55455 USA

**Keywords:** Lasso, GWAS, Resampling, Permutation, Bootstrap, Testing

## Abstract

**Background:**

Genome-wide association studies involve detecting association between millions of genetic variants and a trait, which typically use univariate regression to test association between each single variant and the phenotype. Alternatively, Lasso penalized regression allows one to jointly model the relationship between all genetic variants and the phenotype. However, it is unclear how to best conduct inference on the individual Lasso coefficients, especially in high-dimensional settings.

**Methods:**

We consider six methods for testing the Lasso coefficients: two permutation (Lasso-Ayers, Lasso-PL) and one analytic approach (Lasso-AL) to select the penalty parameter for type-1-error control, residual bootstrap (Lasso-RB), modified residual bootstrap (Lasso-MRB), and a permutation test (Lasso-PT). Methods are compared via simulations and application to the Minnesota Center for Twins and Family Study.

**Results:**

We show that for finite sample sizes with increasing number of null predictors, Lasso-RB, Lasso-MRB, and Lasso-PT fail to be viable methods of inference. However, Lasso-PL and Lasso-AL remain fast and powerful tools for conducting inference with the Lasso, even in high-dimensions.

**Conclusion:**

Our results suggest that the proposed permutation selection procedure (Lasso-PL) and the analytic selection method (Lasso-AL) are fast and powerful alternatives to the standard univariate analysis in genome-wide association studies.

## Background

Genome-wide association studies (GWASs) involve studying association between millions of genetic variants, called “single nucleotide polymorphisms (SNPs)” and different traits of interest. Essentially, a GWAS can be viewed as a high-dimensional variable selection problem with the goal of finding SNPs that are significantly associated with a phenotype of interest. GWASs have predominantly been analyzed using univariate regression, i.e. “single marker association” methods (SMA), where one analyzes the marginal effect between an individual SNP and the phenotype, while ignoring the influence of other SNPs. Assuming that the phenotype is affected by multiple SNPs, SMA neglects useful information regarding the structure of genetic association and hence may lose power to detect relevant SNPs.

Alternatively, jointly modeling all SNPs may lead to more accurate inference due to decreased residual variance in the phenotype of interest. However, given that the number of SNPs greatly exceeds the sample size, standard multiple linear regression techniques are no longer viable. In contrast, penalized regression may be used to jointly estimate regression coefficients in such settings. However, developing valid methods for conducting inference on the penalized regression coefficients remains an open area of research.

Several papers have used penalized regression in GWASs to perform variable selection, without conducting any rigorous inference on the selected variables. For example, Waldmann et al. [1] applied penalized regression to a GWAS by using cross validation to select tuning parameters, then compute the true positive and false positive rates simply based on whether or not a coefficient is nonzero. Cross Validation attempts to optimize the predictive performance of the model, but gives no control on the type-I error rate. Furthermore, both the software PUMA [2] and LOO indices of Wu et al. [3] use penalized regression to perform variable selection, then fit a standard multiple linear regression model using only those variables selected by the initial penalized model. Their software return *p*-values for individual non-zero regression coefficients using standard likelihood-based tests for multiple linear regression. Wu et al. [3] admit that these “pseudo *p*-values” are invalid because they neglect the complex selection procedure of obtaining the reduced model. Although these methods are very fast, they cannot warrant control of the type-I error rate.

One could potentially use resampling techniques to generate correct tests for the individual coefficients in the reduced model. Meinshausen [4] proposed a data-splitting approach to *p*-values with FDR or FWER control. However, sample splitting procedures may be quite conservative compared to methods that directly use the full data [5], likely because they lose power to detect SNPs with smaller effect sizes and minor allele frequencies. Chatterjee and Lahiri [6], and Sartori [7] used cross validation to select penalty parameters, then residual bootstrap confidence intervals (and modifications thereof) to conduct inference on the regression coefficients. Both [6] and [7] focus on bootstrapping the Lasso in low-dimensional settings. Through simulations, we investigate the performance of the bootstrap and modified residual bootstrap when *n* is fixed and the number of null predictors *p*_0_→*∞*.

All previously mentioned methods treat penalty parameter selection and inference as two separate problems. In contrast, Ayers and Cordell [8] proposed a permutation method to select penalty parameters for direct control of the type-1-error rate. If the goal is to detect the genetic variants that meet a certain threshold for the level of significance, Ayers and Cordell [8]’s technique gives us a computationally efficient way of identifying these variants compared to previously mentioned resampling techniques. Similarly, Yi et al. [9] proposed both a permutation and analytic method to select penalty parameters for false-discovery rate control. We propose a modified version of the permutation method of Ayers and Cordell, and compare it with the original method, as well as the analytic method of Yi et al.

Other recently proposed methods of inference for penalized regression not considered in this paper are as follows: Zhang [10] and Javanmard [11] use a “de-biased” Lasso, which attempts to remove the bias in the Lasso coefficients, then constructs normal-based confidence intervals using the transformed coefficients. “Post-selection inference” or “selective inference” [12, 13] is another recent development in inference for penalized regression. Selective inference is described as “the assessment of significance and effect sizes from a dataset after mining the same data to find these associations". For example, suppose one has used Lasso to “select” relevant predictors among a pool of many potentially relevant predictors. In particular, only predictors with nonzero estimated coefficients are considered for inference, all other predictors are dismissed. Selective inference addresses the question of how to conduct valid inference on the subset of selected predictors while accounting for the complex data-dependent procedure that selected those predictors in the first place. In this paper, we decided to focus mainly on resampling-based methods of inference for the Lasso, and thus will not consider the de-biased Lasso or post-selection inferential methods.

Furthermore, one major limitation of existing methods of inference for penalized regression, as they’re currently implemented in the statistical software R [14], is that they require one to store the entire genotype matrix in RAM, thus leading to great computational costs. In contrast, the methods we consider take advantage of the *bigmemory* R package [15] which allows one to work with high-dimensional file-backed datasets that are larger than the available RAM. An important feature of the *bigmemory* package is the ability for multiple cores to share access to the same *big.matrix* object, without having to create an additional copy of the matrix in RAM at each core. Thus the *bigmemory* package allows for memory efficient parallel computing with high-dimensional matrices. In addition, the *biglasso* R package [16] allows one to fit Lasso penalized regression models using the *bigmemory* R package.

Through simulations, we compare six methods for testing the individual Lasso coefficients: two permutation and one analytic selection procedures for type-1-error control, residual bootstrap, modified residual bootstrap, and a permutation test. As a benchmark, all methods are compared to a standard single marker analysis. First we consider the scenario where the sample size (*n*) is fixed, and the number of null SNPs *p*_0_→*∞*. We show that the bootstrap methods and permutation test become unstable as *p*_0_→*∞*; however, both the permutation and analytic selection procedures appear to be powerful tools of inference, even in high-dimensional settings. Lastly, we apply the Lasso with both the permutation and analytic selection methods to the Minnesota Center for Twins and Family Study [17, 18], using 3853 subjects and 507,541 SNPs.

## Methods

Consider a GWAS with *n* subjects, *p* SNPs (*p*>*n*), and a quantitative phenotype *Y*. Without loss of generality, assume there are no additional covariates. Let *x*_*ij*_ denote the number of minor alleles the *i*th subject carries at the *j*th SNP (i.e. *x*_*ij*_=0,1 or 2). Then standardize all SNPs to have a mean of zero and variance of one. We focus on the scenario where the proportion of null SNPs is large, i.e $\frac {p_{0}}{p}\approx 1$, and the individual causal SNPs have relatively small effect sizes. Typically GWASs are analyzed using a “single marker analysis” (SMA), which tests the marginal effect of the *j* ^*th*^ SNP as follows:

1 $$\begin{array}{@{}rcl@{}}  \pmb{Y} = \pmb{x}_{j}\beta_{j}+\pmb{\epsilon}, \ \ \ \pmb{\epsilon}\sim N\left(\pmb{0},\sigma^{2}\pmb{\mathrm{I}}\right) \end{array} $$

Then a t-test may be used to test the null hypothesis H_0_:*β*_*j*_=0. The SMA applies model (1) to all *j*=1,…,*p* SNPs and obtains *p*-values from a t-distribution, then declares a SNP significant if its *p*-value is less than or equal to some desired significance level *α*. In practice, one may adjust for multiple testing by controlling the family-wise error rate or false-discovery rate (see [19] for a review), but we chose not to address the issue of multiple testing in this paper. Rather the aim of this paper is to compare a standard SMA with several multi-marker methods in their ability to detect individual genetic variants that meet a desired level of significance.

As an alternative to SMA, we consider jointly modeling the main effects of all SNPs:

2 $$\begin{array}{@{}rcl@{}}  \pmb{Y}_{n\times 1} = \pmb{\mathrm{X}}_{n\times p}\pmb{\beta}_{p\times 1}+\pmb{\epsilon}_{n\times 1}, \ \ \ \pmb{\epsilon}\sim N\left(\pmb{0},\sigma^{2}\pmb{\mathrm{I}}\right) \end{array} $$

Here we are interested in testing the conditional effect of the *j* ^*th*^ SNP, i.e. the effect of SNP *j* conditional on the effect of all other SNPs. Thus the following null hypothesis is of interest:

3 $$\begin{array}{@{}rcl@{}}  \mathrm{H}_{0}: \ \beta_{j}\big|\pmb{\mathrm{X}_{(-j)}}=0 \end{array} $$

However, given that *p*>*n*, standard multiple linear regression techniques are no longer viable. In contrast, Lasso [20] penalized regression allows one to estimate *β* in high-dimensional settings as follows:

4 $$\begin{array}{@{}rcl@{}} \hat{\pmb{\beta}}_{\lambda}=\underset{\pmb{\beta}}{argmin} \left\{\frac{1}{2}||\pmb{Y}-\pmb{\mathrm{X}}\pmb{\beta}||^{2} + \lambda||\pmb{\beta}||_{1}\right\} \end{array} $$

The subscript *λ* indicates that the Lasso estimator $\hat {\pmb {\beta }}_{\lambda }$ depends on the penalty parameter *λ* which controls the rate of penalization in the estimated coefficients. In general, as *λ* increases, the Lasso coefficients are shrunk closer to zero. Typically Lasso yields a sparse solution of nonzero coefficients, and thus may be viewed as a variable selection tool. When there is a group of highly correlated predictors, the Lasso tends to only select one predictor in the group [21]. It is unclear if this property is disadvantageous for GWAS. If there is a group of highly correlated predictors that represents a causal region and Lasso only detects one SNP in that region; we have detected the region nontheless. A followup analysis could easily find SNPs that are correlated with the detected SNP, if such SNPs would be of interest.

Lastly, most of the methods discussed in this paper can easily be extended to other sparse penalized regression models such as SCAD [22], MCP [23], Elastic Net [24], or Adaptive Lasso [25]. However, it is not the aim of this paper to compare different penalty types, thus we decided to focus only on the Lasso.

### Permutations to select *λ* for type-1-error control

First we describe how to fit a Lasso model using a modified permutation method to select *λ* for control of the type-1-error rate. Define the following decision rule:

5 $$\begin{array}{@{}rcl@{}}  D(\pmb{Y,X},\lambda):= \text{Reject}\ \mathrm{H}_{0}:\beta_{j}=0 \ \text{iff} \ \hat{\beta}_{\lambda,j}\neq0 \end{array} $$

where $\hat {\beta }_{\lambda,j}$ is the estimated Lasso coefficient of the *j* ^*th*^ SNP for a given value of *λ*. Suppose there exists a value of *λ*, called *λ*_*α*_, that controls the type-1-error rate at level *α* under decision rule (5). Ayers and Cordell [8] showed that permutations can be used to estimate *λ*_*α*_. We propose a modified version of Ayers’ method:

1. Permute *Y* to obtain “permuted dataset”: {*Y* ^*P*^,X}. Fit a Lasso model to {*Y* ^*P*^,X}, and then record the value of *λ* that results in exactly *s* nonzero coefficients, where $\frac {s}{p}=\alpha $ and *p* is the total number of predictors. Define this value of *λ* as $\hat {\lambda }_{\alpha }.$
  - Note that if $\alpha < \frac {1}{p}$, the above method won’t work. In this case, suppose we let $\alpha =\frac {1}{p\cdot k}$ for some positive integer *k*. Then we can permute *Y*, *k* additional times (resulting in *k* Lasso models) and record the *λ* value in each of the *k* replicates that results in exactly 1 nonzero coefficient, giving $\{\lambda _{i}\}_{i=1}^{k}.$ Then define $\hat {\lambda }_{\alpha }=max\{\lambda _{i}\}_{i=1}^{k},$ which effectively allows for only 1 nonzero coefficient out of the *k*·*p* total null coefficients, thus controlling the type-1-error rate at level *α*.
  In either case above, $\hat {\lambda }_{\alpha }$ will control the type-1-error rate at approximately level *α*, however, we can reduce the mean squared error of $\hat {\lambda }_{\alpha }$ as follows:
2. Repeat step (1) *B* times to obtain $\{\hat {\lambda }_{\alpha,i}\}_{i=1}^{B}$. Then define the final estimator of *λ*_*α*_ as follows:
  6 $$\begin{array}{@{}rcl@{}}  \bar{\lambda}_{\alpha}=\frac{1}{B}\sum_{i=1}^{B} \hat{\lambda}_{\alpha,i} \end{array} $$
3. Finally, fit the Lasso model to the original data with $\lambda =\bar {\lambda }_{\alpha }$ for type-1-error control at approximately level *α*.

The main modification we propose to Ayers’ method is to estimate *λ*_*α*_ *B* times, then use the sample mean (or median), $\bar {\lambda }_{\alpha }$ as the final estimate of *λ*_*α*_. For high-dimensional datasets, estimating *λ*_*α*_ a single time (as does Ayers and Cordell [8]) may result in an unstable model in terms of variable selection (see Fig. 4 for more details). In contrast, as *B* increases, our estimator $\bar {\lambda }_{\alpha }$ will select a stable model where the number of selected variables converges to some constant. Secondly, we propose two practical modifications to Ayer’s method: 1) use the bisection algorithm of Wu et al. [3] to efficiently find the target *λ*_*α*_, and 2) use the *bigmemory* and *biglasso* R packages for memory-efficient parallel computing with high-dimensional matrices.

Yi et al. [9] proposed a similar permutation and analytic method to select *λ* for false-discovery rate control, which may easily be modified for control of the overall type-1-error rate [5]. Their methods require one to fit a grid of penalty parameter values, with the hope that at least one value on the grid achieves the desired error rate. In contrast, our permutation method will efficiently find the value of *λ* that gives the desired error rate. Through simulations, we compare Lasso using our modified permutation method to select *λ* for type-1-error control (“Lasso-PL”) with the original Ayers’ method (“Lasso-Ayers”), as well as the analytic method of Yi et al. (“Lasso-AL”).

Lastly, we present theoretical justification for using the permutation method to select *λ*.

#### **Theorem 1**

Consider the linear model of the form *Y*=X*β*+*ε*, where X is an *n*×*p* matrix of *p* independent SNPs from *n* independent subjects, each standardized to have a mean of 0 and variance of 1; and *ε*∼*N*(0,*σ* ^2^I_*n*_). Consider the Lasso penalized regression model which can be written in the form of (7), with parameter *λ* that controls the overall rate of penalization. Then under decision rule (5), $\bar {\lambda }_{\alpha }$(6) will control the type-1-error rate at approximately level *α*.

#### *Proof*

Without loss of generality, suppose we wish to control the type-1-error rate at level *α* where $\alpha \ge \frac {1}{p}$ (the proof will have to be modified when $\alpha <\frac {1}{p}$, see step (1a) above for more info). Yi et al. [9] showed that $\beta _{\lambda,j}^{\text {Lasso}}$ can be defined as follows:

7 $$\begin{array}{*{20}l}  \hat{\beta}_{\lambda,j}^{\text{Lasso}} = \left\{ \begin{array}{ll} \hat{\beta}_{j}^{\text{OLS}}-\lambda \ \ \ \text{if}\ \beta_{j}^{\text{OLS}}>\lambda \\ \hat{\beta}_{j}^{\text{OLS}}+\lambda \ \ \ \text{if}\ \beta_{j}^{\text{OLS}}<-\lambda \\ 0 \ \ \ \ \ \ \ \text{if}\ \big|\hat{\beta}_{j}^{\text{OLS}}\big|\le\lambda \end{array} \right. \end{array} $$

where *λ*∈(0,*∞*), and $\hat {\beta }_{j}^{OLS}$ is defined as:

8 $$\begin{array}{@{}rcl@{}}  \hat{\beta}_{j}^{OLS}=\frac{1}{n}\sum_{i=1}^{n}x_{ij}r_{i(j)}, \text{where}\ r_{i(j)}=y_{i} - \underset{k\neq j}{\sum} x_{ik}\beta_{k}. \end{array} $$

The key result of (7) and (8) is that $\hat {\beta }_{\lambda,j}^{\text {Lasso}}$ is nonzero $\text {iff} \ |\hat {\beta }_{j}^{\text {OLS}}|>\lambda.$ Then permuting *Y* approximates the global null scenario: H_0_:*β*=0, which gives the following result:

9 $$\begin{array}{@{}rcl@{}}  \hat{\beta}_{j}^{\text{OLS}}\underset{\mathrm{H}_{0}}{\sim} N\left(0,\frac{\sigma^{2}}{n}\right), \ \ \forall j=1,\ldots,p \end{array} $$

where the asymptotic variance of $\hat {\beta }_{j}^{\text {OLS}}$ is $\frac {\sigma ^{2}}{n}\left (\pmb {x}_{j}^{\intercal } \pmb {x}_{j}\right)^{-1}=\frac {\sigma ^{2}}{n}$ since all covariates are standardized such that $\pmb {x}_{j}^{\intercal }\pmb {x}_{j}=1;$ and *V* *a* *r*(*r*_(*j*)_)=*V* *a* *r*(*Y*)=*σ* ^2^ ∀*j*, since the SNPs explain 0% of the variance in *Y* under the global null.

Next, define the main parameter of interest, *λ*_*α*_, as the $\left (1-\frac {\alpha }{2}\right)\cdot 100\%$ quantile of the distribution of $\hat {\beta }_{j}^{\text {OLS}}$, i.e. $Pr\left (\big |\hat {\beta }_{j}^{\text {OLS}}\big |>\lambda _{\alpha }\right)=\alpha.$ Then we can estimate *λ*_*α*_ as follows:

1. Permute *Y* to obtain *Y* ^*P*^ and fit a Lasso model to {X,*Y* ^*P*^}. Define $\hat {\lambda }_{\alpha }$ as the value of *λ* such that exactly *α* *%* of the penalized $\hat {\beta }_{\lambda,j}^{\text {Lasso}}$’s are nonzero.
2. Then by Eqs. (7) and (8), *α* *%* of the $\hat {\beta }_{\hat {\lambda }_{\alpha },j}^{\text {Lasso}}$’s are nonzero *iff* *α* *%* of the $|\hat {\beta }_{j}^{OLS}|$’s are $>\hat {\lambda }_{\alpha }.$ Therefore $\hat {\lambda }_{\alpha }$ is the $\left (1-\frac {\alpha }{2}\right)\cdot 100\%$ sample quantile estimate of *λ*_*α*_. The asymptotic distribution of the sample quantile estimator $\hat {\lambda }_{\alpha }$ is well known:
  10 $$\begin{array}{@{}rcl@{}}  \sqrt{p}(\hat{\lambda}_{\alpha}-\lambda_{\alpha})\overset{D}{\underset{p\to\infty}{\rightarrow}}N\left(0,\frac{\left(1-\frac{\alpha}{2}\right)\left(\frac{\alpha}{2}\right)}{\phi\left(\frac{\sqrt{n}\lambda_{\alpha}}{\sigma}\right)^{2}}\right) \end{array} $$
  where *ϕ*(·) is the pdf of a *N*(0,1) random variable. Thus $\hat {\lambda }_{\alpha }$ is a consistent estimator for *λ*_*α*_, and will control the type-1-error rate at approximately level *α* for large *p*:
  11 $$ \begin{aligned}  Pr\left(\text{Reject}\ \mathrm{H}_{0}: \beta_{j}=0 | \beta_{j}=0,\hat{\lambda}_{\alpha}\right) &=Pr\left(\hat{\beta}_{\hat{\lambda}_{\alpha},j}^{\text{Lasso}}\neq 0\right)\\ &= Pr\left(\left|\hat{\beta}_{j}^{\text{OLS}}\right|>\hat{\lambda}_{\alpha}\right) \\ &\approx \alpha, \ \ \ \forall j=1,\ldots,p \end{aligned}  $$
  The variability in our estimator can be reduced as follows:
3. Repeat step (1), *B* times to obtain $\big \{\hat {\lambda }_{\alpha,b}\big \}_{b=1}^{B}$ and $\bar {\lambda }_{\alpha }=\frac {1}{B}\sum _{b=1}^{B} \hat {\lambda }_{\alpha,b}$. Then $\bar {\lambda }_{\alpha }\overset {P}{\to }\mathbb {E}(\hat {\lambda }_{\alpha })\approx \lambda _{\alpha }$ for large *p*, and $Var(\bar {\lambda }_{\alpha })=\frac {1}{B}Var(\hat {\lambda }_{\alpha })$; thus $\bar {\lambda }_{\alpha }$ is a more efficient estimator of *λ*_*α*_.

Finally, $\bar {\lambda }_{\alpha }$ will control the type-1-error rate at approximately level *α* for large *p*:

12 $$\begin{array}{*{20}l} Pr(\text{Reject}\ \mathrm{H}_{0}: \beta_{j}=0 | \beta_{j}=0,\bar{\lambda}_{\alpha})&=Pr\left(\hat{\beta}_{\bar{\lambda}_{\alpha},j}^{\text{Lasso}}\neq 0\right) \\ &=Pr\left(\left|\hat{\beta}_{j}^{\text{OLS}}\right|>\bar{\lambda}_{\alpha}\right) \\ &\approx \alpha, \ \forall j=1,\ldots,p \end{array} $$

□

The above theory holds when all SNPs are independent, but may be conservative given dependency among SNPs (see Breheny [5] for more details).

Lastly, we summarize the computational cost of fitting a Lasso-PL model to a real dataset with 3853 subjects and 507,451 SNPs, using a combination of the *glmnet* [26], *bigmemory* [15], and *biglasso* [16] R packages.

1. *Use glmnet to find a reasonable starting window for λ*_*α*_ (≈2.5 min): for a given significance level *α*, this step is a one-time computational cost. It is necessary to find a reasonable window for the target value *λ*_*α*_, in order to speed up computation in step 3. Technically this step could be done with the *biglasso* R package, however, the algorithm we use to find a reasonable starting window for *λ*_*α*_ took around 2.5 min with *glmnet* and over 1 h with *biglasso*. Thus we recommend using *glmnet* for step 1.
2. *Create the big.matrix object* (≈10 min): creating a file-backed *big.matrix* object is a one-time computational cost. In future R sessions, one can instantaneously reload the *big.matrix* object without any overhead.
3. *Fit a biglasso model while estimating λ*_*α*_ *B times*: parallel computing with a base-R *matrix* object would require one to create an additional copy of the matrix within each core, thus leading to high computational cost. In contrast, the *bigmemory* R package allows multiple cores to share access to a single copy of the dataset. Using 20 cores in parallel, this step took around 1 h for *B*=100.

### The residual bootstrap and modified residual bootstrap

For an introduction to the bootstrap, see Efron [27] and Hesterberg [28]. Chatterjee and Lahiri [29] proved that the standard residual bootstrap approximation to the Lasso distribution may be inconsistent whenever one or more components of the regression parameter vector are zero. Chatterjee and Lahiri [6] proposed a “modified residual bootstrap” for the Lasso as an attempt to overcome this problem. However, in low-dimensional settings, Sartori [7] found that the residual bootstrap worked “acceptably well,” and that the modified residual bootstrap appeared to offer no significant improvement. We consider the performance of both the residual bootstrap and modified residual bootstrap when *n* is fixed and *p*_0_→*∞*. The basic setup of the residual bootstrap (“Lasso-RB”) is as follows:

1. Use 10-fold cross-validation to select *λ*, then keep *λ* fixed throughout all remaining steps
2. Fit a Lasso model of the form *Y*=X*β*+*ε* and obtain the Lasso estimate $\hat {\pmb {\beta }}_{\lambda }$
3. Calculate the residuals $\pmb {e}=\pmb {Y}-\hat {\pmb {Y}}$, where $\hat {\pmb {Y}}=\pmb {\mathrm {X}}\hat {\pmb {\beta }}_{\lambda }$. Then center the residuals:
$\pmb {e_{c}}= \pmb {e}-\pmb {\bar {e}}$, where $\bar {\pmb {e}}=\frac {1}{n}\sum _{i=1}^{n} e_{i}$
4. Draw a random sample with replacement of size *n* from the centered residuals, call this $\pmb {e_{c}^{*}}$
5. Define new outcome variable: $\pmb {Y^{*}}=\pmb {\mathrm {X}}\hat {\pmb {\beta }}_{\lambda }+\pmb {e_{c}^{*}}$
6. Fit a Lasso model to {*Y* ^∗^,X,*λ*} and obtain the bootstrap Lasso estimate $\hat {\pmb {\beta }}^{*}_{\lambda }$
7. Repeat *B* times to get $\{\hat {\pmb {\beta }}_{\lambda,b}^{*}\}_{b=1}^{B}$, for large *B*.
8. For all *j*=1,…,*p* SNPs, construct the following bootstrap confidence interval:
$ \text {CI}_{j}=\left (2\hat {\beta }_{\lambda,j} - \hat {\beta }_{\lambda,j,(1-\frac {\alpha }{2})}^{*}, 2\hat {\beta }_{\lambda,j} - \hat {\beta }_{\lambda,j,(\frac {\alpha }{2})}^{*} \right)$, where $\hat {\beta }_{\lambda,j,(\gamma)}$ is the *γ*∗100th quantile of the bootstrap distribution $\{\hat {{\beta }}_{\lambda,j,b}^{*}\}_{b=1}^{B}$
9. For all *j*=1,…,*p* SNPs, reject H_0_: *β*_*j*_=0⇔0∉CI_*j*_

The modified residual bootstrap (“Lasso-MRB”) makes the following changes: step (3) uses the modified bootstrap residuals $\pmb {\tilde {e}}=\pmb {Y}-\pmb {\tilde {Y}}$, where $\pmb {\tilde {Y}}=\pmb {\mathrm {X}}\pmb {\tilde {\beta }}_{\lambda }$, and $\tilde {\beta }_{\lambda,j}=\hat {\beta }_{\lambda,j}\mathbb {I}\big (|\hat {\beta }_{\lambda,j}|>\tau \big)$, for a given threshold *τ*. Step (4) resamples with replacement from these modified centered residuals, call this $\pmb {e_{c}^{**}}$; step (5) uses the new response $\pmb {Y^{**}}=\pmb {\mathrm {X}}\pmb {\tilde {\beta }}_{\lambda }+\pmb {e_{c}^{**}}$, then step (6) fits a Lasso model to {*Y* ^∗∗^,X,*λ*} and obtains the modified bootstrap Lasso estimate $\hat {\pmb {\beta }}^{**}_{\lambda }$. Lastly, step (8) constructs the modified bootstrap confidence interval: $\left (\hat {\beta }_{\lambda,j}+\tilde {\beta }_{\lambda,j}-\hat {{\beta }}_{\lambda,j,(1-\frac {\alpha }{2})}^{**}, \ \hat {\beta }_{\lambda,j}+\tilde {\beta }_{\lambda,j}-\hat {{\beta }}_{\lambda,j,(\frac {\alpha }{2})}^{**}\right).$

### Permutation test *p*-values

Anderson and Legendre [30] provide an overview of permutation tests for multiple linear regression. We extend the permutation test of Manly [31] to the Lasso (“Lasso-PT”) as follows:

1. Use 10-fold cross-validation to select *λ*, then keep *λ* fixed throughout all remaining steps
2. Fit a Lasso model to {*Y*,X,*λ*} and obtain estimate $\hat {\pmb {\beta }}_{\lambda }$
3. Randomly permute *Y* and call it *Y* ^*P*^. Fit the new model *Y* ^*P*^=X*β*+*ε* and obtain the permuted Lasso estimate $\hat {\pmb {\beta }}^{P}_{\lambda }$. Repeat *B* number of times to obtain $\left \{\hat {\pmb {\beta }}^{P}_{\lambda,b}\right \}_{b=1}^{B}$.
4. For an individual predictor *x*_*j*_, calculate the permutation *p*-value:
  $$p_{j}=\frac{\left[\sum_{b=1}^{B} \mathrm{I}\left(|\hat{\beta}_{\lambda,j,b}^{P}| > |\hat{\beta}_{\lambda,j}|\right) \right]+1}{B+1} $$
5. Reject H_0_:*β*_*j*_=0⇔*p*_*j*_≤*α*, where *α* is the specified significance level.

## Results

### Simulations

All simulated genotypes were generated with Vanderbilt’s “GWA Simulator” program [32] using HapMap Illumina300k CEU phased data (Utah Residents with Northern and Western European Ancestry). The GWA simulator can simulate genotypes for case-control designs. We used the simulator to simulate genotypes on a set of markers under the null hypothesis of no association with the disease, and then simulated a quantitative trait on these individuals using linear regression with a set of genetic variants associated with the quantitative trait. Only common variants with minor allele frequencies greater than 0.05 were simulated.

#### Simulation 1

In Simulation 1, we are interested in the scenario where the sample size (*n*) is fixed, the true causal effects are relatively small, and the number of null SNPs *p*_0_→*∞*. Six-hundred subjects were simulated with five independent causal SNPs, each coming from a different chromosome. Without loss of generality, we assume there are no additional covariates. The quantitative trait *Y* was simulated as follows:

13 $$\begin{array}{@{}rcl@{}}  \pmb{Y}_{600\times 1}=\pmb{\mathrm{X}}_{600\times 5} \ \pmb{\beta}_{5\times 1}+\pmb{\epsilon}_{600\times 1}, \ \ \ \pmb{\epsilon}\sim N(\pmb{0},\pmb{\mathrm{I}}) \end{array} $$

After standardizing each SNP to have a mean of zero and variance of one, *β*=0.15J_5_ (where J_5_ is a vector of ones) was chosen so that each causal SNP explains ≈2*%* of the variation in *Y* for a total *R* ^2^≈0.10. Three-hundred datasets were simulated according to (13). Lastly, we consider five different settings where the number of null SNPs varies from 0, 45, 300, 900, and 20,000. The null SNPs were simulated from a chromosome independent of the causal SNPs, and contain varying levels of correlation.

We compare a standard single marker analysis to the Lasso using six different methods of inference on the Lasso coefficients: two permutation and one analytic methods to select *λ* for type-1-error control, residual bootstrap, modified residual bootstrap, and a permutation test. In particular, we are interested in comparing the performance of these methods as the number of null SNPs *p*_0_→*∞*. Lastly, all methods are compared in terms of their true positive rate (TPR): average proportion of causal SNPs detected across the 300 simulated datasets; and false positve rate (FPR): average proportion of null SNPs detected across 300 simulated datasets.

Notice in Table 1 that only Lasso-PL and Lasso-AL consistently have power greater than or equal to the standard SMA. In general, in scenarios where there are not many SNPs, Lasso-AL has slightly greater TPR than Lasso-PL, presumably because Lasso-PL requires a large number of SNPs in order to get a good estimate of *λ*_*α*_ (see proof of Theroem 1). However, in the 20,000 null SNP scenario, Lasso-AL is slightly conservative compared to Lasso-PL.

**Table 1:** Comparison of methods given fixed sample size and increasing number of null SNPs, *α*=0.01

Model	0 Null SNPs	45 Null SNPs	300 Null SNPs	900 Null SNPs	20,000 Null SNPs
Lasso-PL	0.840	0.831 (0.008)	0.841 (0.0081)	0.857 (0.0083)	0.841 (0.0095)
Lasso-AL	0.850	0.850 (0.0102)	0.851 (0.0089)	0.854 (0.0077)	0.828 (0.0079)
Lasso-Ayers	0.819	0.808 (0.0121)	0.836 (0.0096)	0.846 (0.0086)	0.838 (0.0095)
SMA	0.828	0.828 (0.0106)	0.828 (0.0101)	0.828 (0.01)	0.828 (0.01)
Lasso-PT	0.829	0.832 (0.0078)	0.827 (0.0073)	0.818 (0.0076)	0.560 (0.0013)
Lasso-RB	0.869	0.859 (0.0137)	0.847 (0.0106)	0.833 (0.0089)	0.555 (0.0012)
Lasso-MRB(t=0.001)	0.869	0.865 (0.0138)	0.850 (0.0105)	0.838 (0.0089)	0.556 (0.0012)
Lasso-MRB(t=0.005)	0.869	0.863 (0.0137)	0.849 (0.0105)	0.836 (0.009)	0.555 (0.0012)
Lasso-MRB(t=0.01)	0.869	0.864 (0.0139)	0.849 (0.0105)	0.837 (0.009)	0.556 (0.0012)
Lasso-MRB(t=0.03)	0.869	0.864 (0.0136)	0.849 (0.0102)	0.839 (0.0092)	0.558 (0.0013)
Lasso-MRB(t=0.05)	0.869	0.859 (0.0124)	0.841 (0.0099)	0.833 (0.0089)	0.559 (0.0013)

Models are compared by their true positive rate and false positive rate (in parentheses), across 300 simulated datasets, using a significance level of *α*=0.01. Each column represents a scenario where a different number of null SNPs were used (e.g. 0, 45, 300, 900, or 20,000)

In our simulation study, Lasso-Ayers is consistently less powerful than Lasso-PL (especially in the 0 and 45 null SNP scenario), and has slightly inflated type-1-error in the 45 null SNP scenario. A Wilcoxon signed rank test to assess if the average false-positive rate (across 300 datasets) for Lasso-Ayers differs from the expected type-1-error rate *α*=0.01 produced a *p*-value of 0.016. Because Lasso-Ayers only estimates *λ*_*α*_ a single time, there may be high variability in $\hat {\lambda }_{\alpha }$ compared to Lasso-PL which estimates *λ*_*α*_ *B* times then uses the sample mean (or median) as its final estimate (this can clearly be seen in Table 2). Thus on average, Lasso-Ayers is more prone to missing potential causal SNPs by over-estimating *λ*_*α*_, or under-estimating *λ*_*α*_ and having excess false positives. Lasso-PL appears to correct this by obtaining a more stable estimate of *λ*_*α*_, thus having increased power and better control of the type-1-error relative to Lasso-Ayers.

**Table 2:** Comparison of $\pmb {\hat {\lambda }_{\alpha }}$ between Lasso-Ayers, Lasso-PL, and Lasso-AL

Model	0 Null SNPs	45 Null SNPs	300 Null SNPs	900 Null SNPs	20,000 Null SNPs
Lasso-Ayers	0.112 (0.0168)	0.112 (0.0165)	0.108 (0.0099)	0.105 (0.0065)	0.074 (0.0031)
Lasso-PL	0.110 (0.0042)	0.110 (0.0041)	0.109 (0.0034)	0.105 (0.0033)	0.074 (0.0022)
Lasso-AL	0.108 (0.0033)	0.107 (0.0033)	0.107 (0.0033)	0.106 (0.0033)	0.081 (0.0039)

The average selected value of *λ* that controls the type-1-error rate at level *α*=0.01 is compared between three different methods across 300 simulated datasets. Standard deviations are reported in parentheses

Table 2 presents a comparison of $\hat {\lambda }_{\alpha }$ from Lasso-Ayers, Lasso-PL, and Lasso-AL. Notice that Lasso-Ayers and Lasso-PL, on average, select the same value of *λ*; however, Lasso-PL significantly reduces the variability in $\hat {\lambda }_{\alpha }$, and thus may provide more accurate inference. Although the variability in $\hat {\lambda }_{\alpha }$ may appear small for all methods, the scale is relative, for small changes in *λ* can result in drastically different number of nonzero coefficients, especially in high-dimensional settings. In the low dimensional settings, the three different methods produce similar values of $\hat {\lambda }_{\alpha }$ on average, with Lasso-Ayers being the most variable. In the high-dimensional 20,000 null SNP scenario, Lasso-AL appears to be slightly over-penalizing relative to Lasso-PL and Lasso-Ayers.

When there is no null SNP, Lasso-RB and Lasso-MRB are the most powerful models; but when there are 45 null SNPs, all of the bootstrap methods have significantly inflated FPR. The reason the FPR is inflated may be due to the theoretical work of [6, 29] which shows that the bootstrap may become unstable given one or more null predictors. In addition, notice as the number of null SNPs increases, the bootstrap methods become increasingly conservative, such that the power in the 20,000 null SNP scenario is significantly less than the other competing methods.

**Fig. 1:**
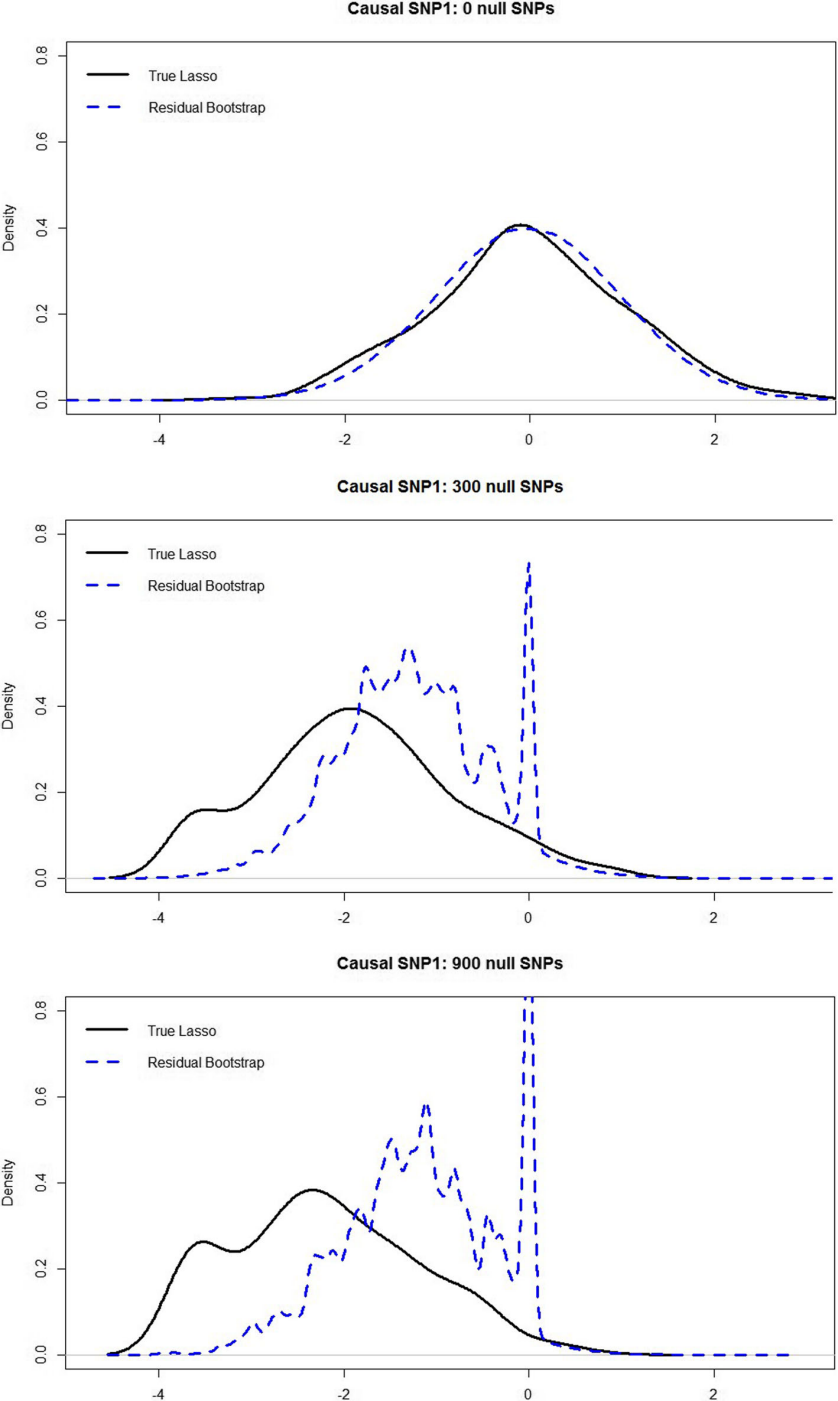
Comparison of the true Lasso distribution with the residual bootstrap approximation of the Lasso distribution. The *black curve* represents the empirical “true” Lasso distribution of $T_{1}=\sqrt {n}(\hat {\beta }_{\lambda,1}-\beta _{1})$, over 300 simulated datasets. The *blue curve* combines the residual bootstrap distribution of $T_{1}^{*}=\sqrt {n}(\hat {\beta }^{*}_{\lambda,1}-\hat {\beta }_{\lambda,1})$ from all 300 datasets

**Fig. 2:**
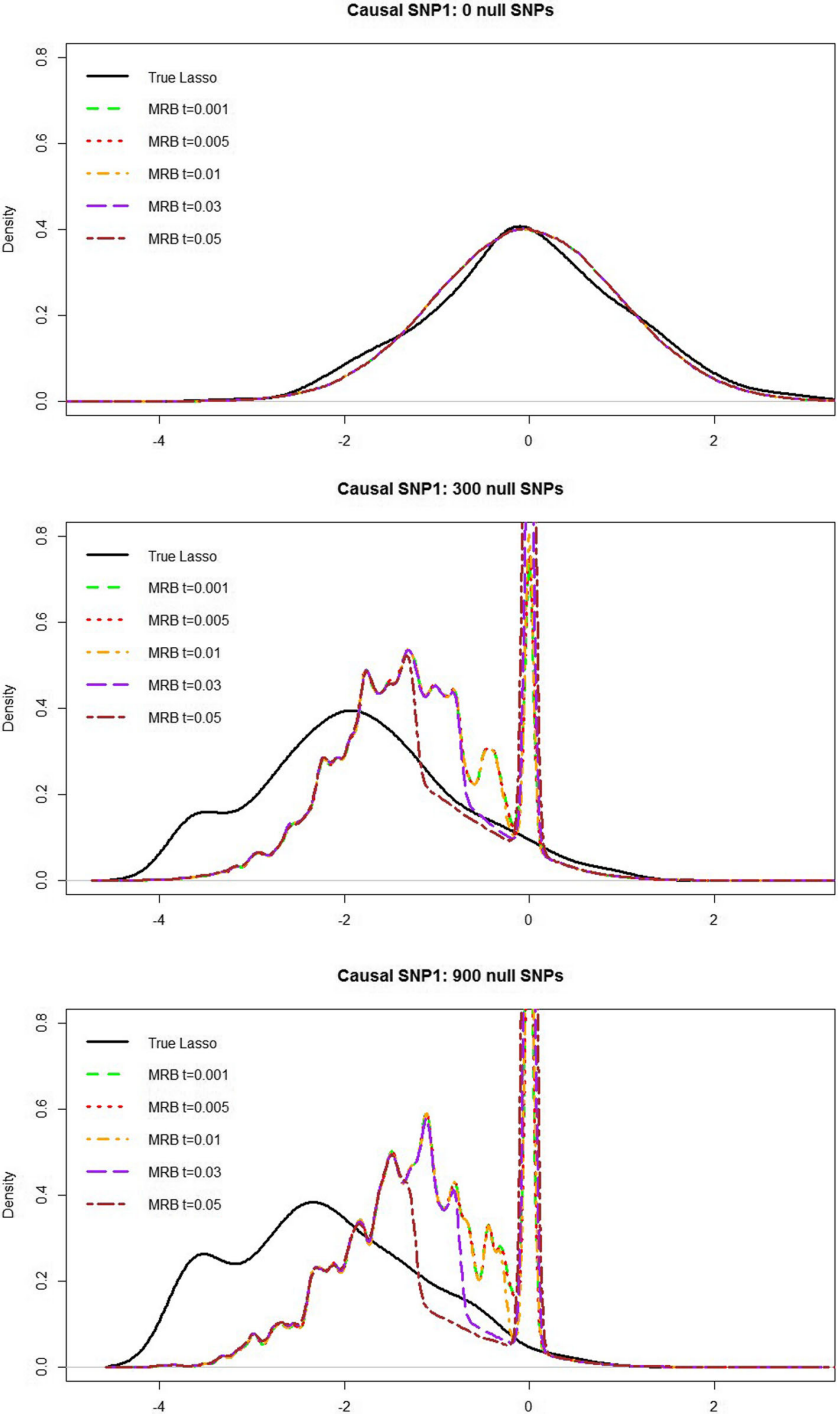
Comparison of the true Lasso distribution with the modified residual bootstrap approximation to the distribution of $\hat {\beta }_{\lambda,1},$ with increasing number of null SNPs. The *black curve* represents the empirical “true” Lasso distribution of $T_{1}=\sqrt {n}(\hat {\beta }_{1}-\beta _{1})$, over 300 simulated datasets. The other curves combine the modified residual bootstrap distribution of $T_{1}^{**}=\sqrt {n}(\hat {\beta }^{**}_{\lambda,1}-\tilde {\beta }_{\lambda,1})$ from all 300 datasets

Notice Figs. 1 and 2 show that the bootstrap methods perfectly approximate the true Lasso distribution when there are no null SNPs. However, as *p*_0_ increases, the bootstrap methods area no longer able to approximate the true Lasso distribution; thus hypothesis testing using the bootstrap may fail to control the type-1-error at the correct level (as seen in the 45 and 20,000 null SNP scenarios). In addition, notice that the modified residual bootstrap is unable to provide significantly better approximations to the true Lasso distribution, compared to the standard residual bootstrap in Fig. 1. We tried a range of threshold values for the Lasso-MRB: *t*=0.001,0.005,0.01,0.03, and 0.05. For values of *t*≤0.001, the MRB performed nearly identical to the RB, and for *t*>0.05 the MRB degenerates to a large point mass at zero. However, for all *t*∈(0.001,0.05), the MRB does not appear to significantly improve upon the standard residual bootstrap.

Similar to the bootstrap methods, the permutation test (Lasso-PT) becomes significantly conservative in the setting with 20,000 null SNPs. One reason why the bootstrap and permutation test perform so poorly as *p*_0_ increases may be because using 10-fold CV to select *λ* in high-dimensional settings is too stringent, resulting in over-penalization of the coefficients.

According to Table 3, as the number of null SNPs increases, the average *λ* selected by 10-fold CV increases and gets very close to the true effect size of the causal SNPs (0.15). Thus using 10-fold CV to select *λ* in high-dimensional settings may be too stringent, resulting in over-penalization of the coefficients, and thus lead to conservative results. In the 20,000 null SNP setting, even in the best case scenario where if every non-zero coefficient was declared significant, Lasso-PT and the bootstrap methods would still all have TPR less than 0.6 (which is much less than the competing methods). Thus clearly the 10-fold CV is over-penalizing the coefficients and making it harder to distinguish the causal SNPs from the null SNPs in this setting.

**Table 3:** Average *λ* selected by 10-fold cross validation

Num. of Null SNPs	Avg. *λ* (Std. dev.)
0	0.0007 (0.0013)
45	0.048 (0.011)
300	0.076 (0.013)
900	0.089 (0.015)
20000	0.136 (0.024)

For Lasso-RB, Lasso-MRB, and Lasso-PT: the average *λ* selected by 10-fold CV across 300 simulated datasets is reported for simulation scenarios with varying number of null SNPs. Standard deviations are listed in parentheses

Given that the performance of Lasso-RB, Lasso-MRB, and Lasso-PT significantly degenerates as *p*_0_ increases (especially in the 20,000 null SNP scenario), we decided to omit these methods from the remainder of our paper.

### Simulation 2

Simulation 2 is similar to Simulation 1, except now we allow each causal SNP to be correlated with several neighboring SNPs. Specifically, each causal SNP was allowed ten neighboring SNPs in varying levels of linkage-disequilibrium (LD) with the causal SNP. Thus we have five causal regions or “LD blocks” of SNPs that are associated with the disease trait. Kruglyak [33] and Pritchard [34] defined “useful LD” as having *r*>0.316 or *r* ^2^>0.1, where *r* is defined in [35]. Thus when picking the LD regions, we tried to ensure that each region had several representative SNPs in “useful LD” with that region’s causal SNP. A summary of each causal region’s LD structure is as follows: in region 1, the average *r* ^2^ between the 10 neighboring SNPs with the true causal SNP was 0.18, with 3 SNPs having an *r* ^2^>0.4 with the causal SNP (max=0.52). In region 2, the average *r* ^2^ was 0.24, with 4 SNPs having an *r* ^2^>0.3 with the causal SNP (max=0.8). In region 3, the average *r* ^2^ equaled 0.29, with 3 SNPs having an *r* ^2^>0.4 with the causal SNP (max=0.65). In region 4, the average *r* ^2^ equaled 0.33, with 4 SNPs having an *r* ^2^>0.4 with the causal SNP (max=0.81). In region 5, the average *r* ^2^ was 0.32, with 3 SNPs having an *r* ^2^>0.4 with the causal SNP (max=0.67). Thus overall, each causal region contains multiple SNPs that “represent” the region by having moderate to large LD with the latent causal SNP. Lastly, the five original causal SNPs were removed. Thus the goal is to “detect” a causal region by detecting at least one SNP that is in significant LD with the latent causal SNP of that region. In reality, the true causal SNPs are often not sequenced, thus our goal is to “tag” the true causal SNP by detecting SNPs in significant LD with the latent causal SNP. In each simulated dataset, there are 1000 total SNPs. A “null” SNP is defined as any SNP that is *not* in significant LD with any of the five latent causal SNPs. We defined a SNP as being in “significant LD” with a latent causal SNP if *r*≥*τ*, and considered two different values for *τ*: 0.3 and 0.5.

Models are compared by their true positive rate (TPR), linked true positive rate (LTPR), and false positive rate (FPR). The TPR represents the average proportion of the five causal regions that are detected across the three-hundred simulated datasets. To detect a causal region, one must detect *at least one* SNP that is in significant LD with that region’s latent causal SNP. The LTPR is defined as the average proportion of SNPs detected that are in significant LD with at least one latent causal SNP. For example, in a given dataset, suppose there are 20 SNPs that are in significant LD with at least one latent causal SNP; and suppose a model detects 10 of these SNPs as having significant association with the disease trait. This would result in an LTPR of $\frac {10}{20}=0.5$. In contrast, suppose I detect at least 1 SNP in 3 of the 5 causal regions that is in significant LD with that region’s latent causal SNP. This would result in a TPR of $\frac {3}{5}=0.6.$

Lastly, FPR(*τ*) represents the average proportion of null hypotheses that are falsely rejected, where a null SNP is defined as not being in significant LD (using cutoff *τ*) with any latent causal SNP.

Notice in Table 4 that Lasso-PL, Lasso-Ayers, and Lasso-AL have higher TPR than SMA for both cutoffs *τ*=0.3 and 0.5. Recall here that TPR measures the ability of a model to detect the true latent causal SNPs. Secondly, notice that SMA has significantly higher LTPR than the Lasso models. We should expect this because given a group of highly correlated SNPs that are also correlated with a latent causal SNP, Lasso tends to only select one SNP within the group, whereas SMA is likely to select multiple SNPs. However, given that the Lasso models have higher TPR than SMA implies that many of the SNPs SMA is picking up are redundant and not offering much additional information about the latent causal SNP.

**Table 4:** Comparison of TPR, LTPR, and FPR across 300 simulated datasets, each with five causal regions

Model	TPR(t=0.3)	LTPR(t=0.3)	FPR(t=0.3)	TPR(t=0.5)	LTPR(t=0.5)	FPR(t=0.5)
Lasso-PL	0.780	0.166	0.0087	0.762	0.232	0.0091
Lasso-Ayers	0.775	0.166	0.0092	0.755	0.232	0.0096
Lasso-AL	0.761	0.159	0.0075	0.741	0.222	0.0079
SMA	0.753	0.336	0.0101	0.740	0.448	0.0115

Each dataset contains *n*=600 subjects, 1000 SNPs and five causal regions. All testing was done using significance level *α*=0.01. See Section “Simulation 2” for a definition of “causal region”, TPR, LTPR, and FPR

For a given correlation threshold *τ*, the penalized regression models appear to do a better job of controlling the FPR at level *α*. This is probably because SMA is more likely to detect spurious SNPs that are weakly correlated with the latent causal SNPs.

Interestingly, there appears to be negligible difference between Lasso-PL and Lasso-Ayers in Simulation 2; whereas in Simulation 1, Lasso-PL was consistently more powerful and better controlled the type-1-error rate. However, given the results from Simulation 1 and our real data analysis, Lasso-PL appears to be the better method despite the increased computational cost.

### Minnesota Center for Twins and Family Study (MCTFS)

The Minnesota Center for Twins and Family Study [17, 18] contains genotype information on over 520,000 SNPs using Illumina’s Human 660W Quad Array, with 8405 subjects clustered into 4-member families (each with 2 parents and 2 children). The families are categorized by sibling relationship type: MZ twins, DZ twins, full siblings, adopted siblings, and mixed siblings (one adopted, one biological). The overall goal of the study is to explore the genetic and environmental factors of substance abuse.

After quality control procedures, we focused on 3853 caucasian parents and 507,541 SNPs with MAF >1*%*, HWE *p*-values >10^−6^, and genotype call rates >99*%* (see [18] for more details). Remaining missing genotypes were imputed using a combination of Beagle [36] and minimac [37], since existing penalized regression software cannot handle missing data.

We decided to focus on two quantitative clinical phenotypes created by [18], which were derived using the hierarchical factor analytic approach of [38]. These 2 phenotypes of interest are: (1) Alcohol Consumption (composite of measures of alcohol use frequency and quantity); and (2) Non-Substance Behavioral Disinhibition (composite of measures non-substance use behavioral disinhibition including symptoms of conduct disorder and aggression). For each phenotype, we first fit a mixed linear model with covariates: Sex, Age, top 10 principle components, and a random intercept for Family ID. In this case, two spouses are given the same Family ID. The random intercept is included because spouses may exhibit correlated substance abuse behavior. The conditional residuals (that account for both fixed and random effects) from this fit were used as the new response for all subsequent genetic testing. Since Lasso-PL can only use significance levels of the form $\alpha =\frac {s}{p}$ or $\frac {1}{p*k}$ for positive integers *s* and *k*, we used the significance level $\alpha =\frac {6}{507,541}\approx 1.18*10^{-5}$ for all tests. In practice, one would choose *α* *apriori* (e.g. 10^−5^), then pick *s* or *k* to get as close to the desired *α* level as possible.

**Fig. 3:**
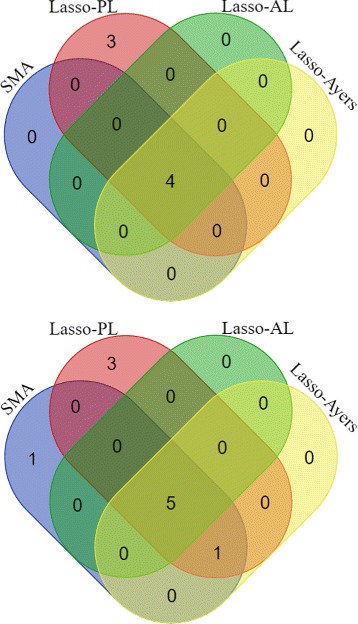
Comparison of selected number of SNPs in a GWAS with two different quantitative traits: alcohol consumption (*top*) and non-substance behavioral disinhibition (*bottom*). A total of 3853 subjects were used with 507,541 SNPs. All testing used significance level *α*=1.18∗10^−5^. Venn Diagrams were created using [39]

Lastly, for each phenotype, we compared Lasso-PL (with *B*=100), Lasso-AL, and Lasso-Ayers to the standard single marker analysis (SMA). Results for the GWAS of Alcohol Consumption and Non-Substance Behavioral Disinhibition can be found in Fig. 3 and Tables 5-6.

**Fig. 4:**
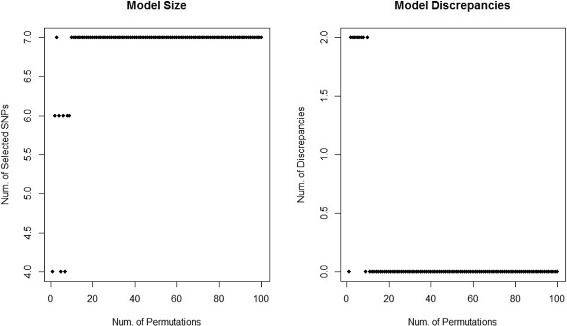
Diagnostic plots for Lasso-PL with the alcohol consumption quantitative trait. The figure on the *left* shows the total number of selected SNPs for a given number of permutations (*B*) used in the estimator $\bar {\lambda }_{\alpha }.$ Ideally, as the number of permutations increases, the number of selected SNPs should converge to some constant $\left (\text {since } Var\left (\bar {\lambda }_{\alpha }\right)\mathop {\rightarrow }\limits _{B\to \infty } 0\right)$. The figure on the *right* shows the number of discrepant SNPs between models using *B* and *B*−1 permutations in the estimator $\bar {\lambda }_{\alpha }$. Ideally, as *B* increases, the number of discrepant SNPs should converge to 0

**Table 5:** Alcohol Consumption GWAS

SNP	Chr.	Gene	Distance from Gene (bp)	SMA	Lasso-PL	Lasso-AL	Lasso-Ayers
rs7574612	2	LHCGR	-24421	9.4∗10^−6^ (S)	S	S	S
rs4836266	5	GRAMD3	-235	1.3∗10^−5^ (N)	S	N	N
rs211598	6	EYA4	-29329	9.3∗10^−6^ (S)	S	S	S
rs4385434	8	hCG_1814486	-129857	5.5∗10^−6^ (S)	S	S	S
rs7136989	12	GOLGA3	-244	1.7∗10^−5^ (N)	S	N	N
rs6072694	20	PTPRT	-1180	2.1∗10^−6^ (S)	S	S	S
rs233278	21	KRTAP10-4	-1677	1.3∗10^−6^ (N)	S	N	N

For Tables 5 and 6: “S” means ”significant” and “N” means “not significant” using significance level *α*=1.18∗10^−5^. Note that Lasso-PL, Lasso-AL, and Lasso-Ayers cannot provide exact *p*-values, but selects significant SNPs while attempting to control the type-1-error rate at level *α*. One could fit multiple penalized regression models and estimate *λ* that controls the type-1-error rate at various orders of magnitude (e.g. 10^−5^,10^−6^, etc) to get a better idea of the significance of each selected SNP (not done here)

**Table 6:** Non-substance behavioral disinhibition GWAS

SNP	Chr.	Gene	Distance from Gene (bp)	SMA	Lasso-PL	Lasso-AL	Lasso-Ayers
rs831750	1	LOC440706	-1546	8.1∗10^−6^ (S)	N	N	N
rs1007227	1	LOC440706	-698	7.5∗10^−7^ (S)	S	S	S
rs17045125	2	ASB3	-3399	1.3∗10^−5^ (N)	S	N	N
rs1384394	2	IKZF2	-32475	7.9∗10^−6^ (S)	S	S	S
rs4527483	4	TSPAN5	-7088	6.9∗10^−6^ (S)	S	S	S
rs3017726	4	LOC728847	-88224	1.3∗10^−5^ (N)	S	N	N
rs6923361	6	MCHR2	-58727	5.0∗10^−6^ (S)	S	S	S
rs2215987	7	THSD7A	-177262	1.4∗10^−5^ (N)	S	N	N
rs10504658	8	PXMP3	-718715	1.3∗10^−6^ (S)	S	S	S
rs7314533	12	KCNC2	-81891	9.7∗10^−6^ (S)	S	N	S

Notice for the GWAS of Alcohol Consumption (Table 5) that SMA, Lasso-AL, and Lasso-Ayers detected only 4 SNPs, whereas Lasso-PL detected 7 SNPs. For the GWAS of Non-Substance Behavioral Disinhibition (Table 6), SMA detected 7 SNPs, while Lasso-PL detected 6 of the 7 SNPs found by SMA, plus 3 additional SNPs. Note the only SNP found by SMA that was not found by Lasso-PL (rs831750), is significantly correlated with SNP rs1007227 (*r* ^2^=0.788), which was detected by all models. However, Lasso-AL failed to tag SNP rs7314533 which was detected by all other models. Thus there appears to be slight evidence that Lasso-PL and Lasso-Ayers are more powerful than Lasso-AL here. Secondly, the few SNPs that Lasso-PL detected that SMA missed, were still borderline significant for SMA. Thus overall, the three Lasso models performed very similar to the standard SMA, with evidence that Lasso-PL may be slightly more powerful, and Lasso-AL may be conservative. As expected, if there are two highly correlated SNPs that are associated with the trait of interest, SMA is more likely to detect both SNPs compared to penalized regression; but Lasso-PL and Lasso-Ayers are still detecting the associated region nontheless.

A natural question is whether *B*=100 permutations is sufficient for obtaining an accurate estimate of *λ*_*α*_ in the Lasso-PL models. We created a diagnostic tool to assess whether or not *B* is large enough (see Fig. 4).

In order to indicate that we have obtained a stable model in terms of variable selection (i.e. that $\bar {\lambda }_{\alpha }$ is an accurate estimate of *λ*_*α*_), we need the number of selected SNPs to converge to some constant and the number of discrepant SNPs to converge to 0, as the number of permutations →*∞*. In Fig. 4, notice as the number of permutations used in the estimator $\bar {\lambda }_{\alpha }$ increases, the number of SNPs our model selects converges to 7; and the number of discrepancies in selected SNPs between models using *B* and *B*−1 permutations converges to 0. Thus it appears that using at least 15 permutations is sufficient for obtaining a stable estimate of *λ*_*α*_ in this application. Note if we had estimated *λ*_*α*_ only a single time, as does Ayers and Cordell [8], we would have identified only four nonzero SNPs, thus missing the three potentially associated SNPs that $\bar {\lambda }_{\alpha }$ identifies when *B*>15. Overall, *B*=100 was more than enough to ensure an accurate estimate of *λ*_*α*_ for the two Lasso-PL models.

Lastly, a comparison of the computation time required to fit each model can be found in Table 7. Notice that all of the methods considered in this paper have reasonable computational costs for a realistic large-scale GWAS, with SMA and Lasso-Ayers being the fastest methods. Although Lasso-AL is an analytical method, it still requires one to fit a Lasso model using a grid of *λ* values, then estimates the type-1-error rate for each value of *λ* analytically, with the hope that at least one value of *λ* within the grid obtains an estimated type-1-error rate near the desired level *α*. This calculation cannot be done *apriori* because it requires estimation of *σ* ^2^ in Eq. 2 for each value of *λ* in the grid. We attempted to find a reasonable window for the target value of *λ* beforehand, then fit a grid of *λ* values within this window in order to reduce the computational cost. However, it may be possible to further reduce the computational time needed to fit Lasso-AL models by picking a more optimal grid. Lastly, notice the computation time for Lasso-Ayers with the Behavioral Disinhibition trait is almost twice the time needed for the Alcohol Consumption trait. This is because the bisection algorithm [3] we used to find the target value of *λ* (in both Lasso-Ayers and Lasso-PL models) sometimes has high variability in computation time. Thus it may be possible to further reduce the computation time needed to fit Lasso-Ayers or Lasso-PL by developing more efficient algorithms for finding the target value of *λ*. However, our current implementation of these methods seems reasonable enough using modest computational resources.

**Table 7:** Computation time in minutes for alcohol consumption and behavioral disinhibition GWASs

Method	Cores	Alc_CON	Behav_Dis
SMA	20	8.0	7.9
Lasso-Ayers	1	10.5	19.9
Lasso-AL	1	29.2	29.4
Lasso-PL(*B*=100)	20	57.1	62.0

Computation time in minutes for the models fit to the Alcohol Consumption and Behavioral Disinhibition quantitative traits, using 507,541 SNPS from 3853 subjects

## Discussion

Penalized regression is a useful tool for GWASs that allows one to simultaneously test the relationship between hundreds of thousands of SNPs and a phenotype of interest with a single model. Unlike the standard single marker analysis, penalized regression jointly models all SNPs, and thus provides a more realistic model of the structure of genotype-phenotype association.

Through simulations, we compared six methods for conducting inference on the individual Lasso coefficients: two permutation and one analytic approachs to select *λ* for type-1-error control, residual bootstrap, modified residual bootstrap, and permutation test *p*-values. Simulation 1 showed that for fixed sample size and increasing number of null SNPs, the bootstrap methods fail to approximate the true Lasso distribution. In addition, the modified residual bootstrap gave no significant advantage over the standard residual bootstrap. In the simulation scenario with 20,000 null SNPs, the bootstrap methods (Lasso-RB, Lasso-MRB) and permutation test (Lasso-PT) become significantly conservative relative to competing methods of inference. Therefore, we do not recommend using the residual bootstrap, modified residual bootstrap, or permutation test with the Lasso in high-dimensional settings.

Throughout our simulations, we found using our modified permutation approach or an analytic method to select *λ* for type-1-error control (Lasso-PL and Lasso-AL) were often the most powerful models, with power consistently greater than or equal to the standard SMA. Unlike the bootstrap or permutation test, Lasso-PL and Lasso-AL performed consistently well, even in high-dimensional settings. As to which method, Lasso-PL or Lasso-AL is more powerful, our study gave mixed results. In Simulation 1, Lasso-AL was more powerful in low-dimensional settings, but Lasso-PL was more powerful in the 20,000 null SNP setting. In Simulation 2 and our real data analysis, Lasso-PL was more powerful. For the real data GWAS of Alcohol Consumption, Lasso-AL failed to tag a SNP detected by the standard SMA, while both Lasso-PL and Lasso-Ayers successfully tagged this SNP. Overall, both Lasso-PL and Lasso-AL were consistently competitive with the standard SMA, thus it seems either method could be recommended in practice.

Simulation 1 and the real data application gave evidence that our modified permutation method to select *λ* (Lasso-PL) performs better than the original method (Lasso-Ayers) proposed by Ayers and Cordell [8]. Lasso-Ayers uses permutations to estimate the value of *λ* that controls the type-1-error rate at the desired level *α*. They use permutations to estimate the target *λ*_*α*_ only a single time, whereas Lasso-PL estimates *λ*_*α*_ *B* times then uses the sample mean (or median) as its final estimate, thus reducing the variability in $\hat {\lambda }_{\alpha }.$ Simulation 1 showed that Lasso-PL is consistently more powerful than Lasso-Ayers, and does a better job of controlling the type-1-error rate. Table 2 showed that Lasso-PL significantly reduces the variability in $\hat {\lambda }_{\alpha }$ compared to Lasso-Ayers. In addition, Lasso-PL always detected more SNPs in the real data analysis. By using permutations to estimate *λ*_*α*_ only a single time, Lasso-Ayers is more prone to missing potential causal SNPs by over-estimating *λ*_*α*_, or under-estimating *λ*_*α*_ and having excess false-positives. Lasso-PL corrects this by obtaining a more stable estimate of *λ*_*α*_.

Another key difference between Lasso-Ayers and Lasso-PL, is that Lasso-PL should lead to more consistent results. For example, if multiple Lasso-Ayers models are fit to the same high-dimensional dataset, there may be high variability in the number of selected relevant SNPs between each model. Whereas if multiple Lasso-PL models are fit to the same dataset, they should all obtain the same results given a sufficient number of permutations.

The main downsides of Lasso-PL, Lasso-AL, and Lasso-Ayers is that they provide no confidence intervals or exact *p*-values for individual SNPs. We are still guaranteed that the subset of selected SNPs maintains approximate type-1-error control at level *α*, but do not know exactly “how significant” each selected SNP is. One could fit multiple Lasso-PL or Lasso-AL models and estimate *λ* that controls the type-1-error rate at various orders of magnitude (e.g. 10^−5^,10^−6^, etc) to get a better idea of the significance of each selected SNP, however, this would greatly increase the computational cost. Nonetheless, if our goal is to identify the genetic variants that meet a pre-specified level of significance, then Lasso-PL and Lasso-AL are fast and powerful alternatives to the standard single marker analysis.

Lastly, throughout this paper the methods we use control the overall type-1-error rate. However, these methods can easily be modified for control of the family-wise-error rate at level *α* by using the significance level $\alpha ^{*}= \frac {\alpha }{p}$ where *p* is the total number of SNPs or estimated number of effective tests. For controlling the false-discovery rate with penalized regression, see [9].

## Conclusion

Developing valid methods to test Lasso coefficients in high-dimensional settings remains a challenging area of research. Through simulations, we’ve shown that the residual bootstrap (Lasso-RB), modified residual bootstrap (Lasso-MRB), and permutation test (Lasso-PT) become practically intractable in high-dimensional settings (*p*>>*n*). However, our modified permutation method to select *λ* for type-1-error control (Lasso-PL) and the analytic method of Yi et al. [9] (Lasso-AL) nearly always outperformed the standard univariate analysis in both simulations and real data application. The *bigmemory* and *biglasso* R packages may be used to fit high-dimensional Lasso-PL or Lasso-AL models with memory-efficient parallel computing. For a real dataset with 3853 subjects and 507,451 SNPs, Lasso-PL with *B*=100 permutations took around one hour using 20 cores in parallel, while Lasso-AL took less than 30 min with a single core. Therefore, we recommend Lasso-PL or Lasso-AL as fast and powerful alternatives to the standard single marker analysis in genome-wide association studies.
